# Two types of social grooming methods depending on the trade-off between the number and strength of social relationships

**DOI:** 10.1098/rsos.180148

**Published:** 2018-08-01

**Authors:** Masanori Takano

**Affiliations:** Akihabara Laboratory, CyberAgent, Inc., Chiyoda-ku, Tokyo, Japan

**Keywords:** social networking site, primitive communications, modern communications, socialgrooming, weak ties, social relationship from

## Abstract

Humans use various social bonding methods known as social grooming, e.g. face to face communication, greetings, phone and social networking sites (SNS). SNS have drastically decreased time and distance constraints of social grooming. In this paper, I show that two types of social grooming (elaborate social grooming and lightweight social grooming) were discovered in a model constructed by 13 communication datasets including face to face, SNS and Chacma baboons. The separation of social grooming methods is caused by a difference in the trade-off between the number and strength of social relationships. The trade-off of elaborate social grooming is weaker than the trade-off of lightweight social grooming. On the other hand, the time and effort of elaborate methods are higher than those of lightweight methods. Additionally, my model connects social grooming behaviour and social relationship forms with these trade-offs. By analysing the model, I show that individuals tend to use elaborate social grooming to reinforce a few close relationships (e.g. face to face and Chacma baboons). By contrast, people tend to use lightweight social grooming to maintain many weak relationships (e.g. SNS). Humans with lightweight methods who live in significantly complex societies use various types of social grooming to effectively construct social relationships.

## 1. Introduction

The behaviour of constructing social relationships is called ‘social grooming’, which is not limited to humans but widely observed in primates [1–9]. Humans use different social grooming methods according to their strength of social relationships [9,10] (see also electronic supplementary materials, figure S1), e.g. primitive methods (face-to-face communications) and modern methods (e-mails and social networking sites (SNS)). Social grooming gives different impressions and has different effects on its recipients depending on the time and effort involved [11]. Face-to-face communication and on video calls get more satisfaction than communication in phone and text [12]. On Facebook, personal messages give more happiness than 1-click messages (like) and broadcast messages [13]. In other words, humans favour social grooming by elaborate methods (time-consuming and space constrained). Additionally, people in a close relationship tend to do these elaborate methods [10]. Furthermore, its positive effect in close relationships is larger than in weak social relationships [13].

Humans face cognitive constraints [14] (for example, memory and processing capacity) and time constraints (that is, time costs) in constructing and maintaining social relationships. These time costs are not negligible, as humans spend a fifth of their day in social grooming [15] and maintaining social relationships [16,17]. Therefore, the mean strength of existing social relationships has a negative correlation with the number of social relationships [9,18,19]. The trade-off between the number *N* and mean strength *m* of social relationships on online communications (SNS, mobile phones and SMS) is described as *C* = *Nm*^*a*^ where *a* > 1 [9], i.e. total communication cost *C* obeys *Nm*^*a*^. *C* which represents the amount of investment for social relationships differs depending on individuals. This suggests that social grooming behaviour depends on the strength of social relationships and the strength of this trade-off (*a*).

Humans construct and maintain diverse social relationships within the constraints of this trade-off. These relationships provide various advantages to them in complex societies. Close social relationships lead to mutual cooperation [7,8,20,21]. On the other hand, having many weak social relationships, i.e. weak ties, helps in obtaining information, which is advantageous because weak social relationships where people rarely share knowledge often provide novel information [3,22–25].

As a result, social relationship forms (distributions of social relationship strengths) often show a much skewed distribution [26,27] (distributions following a power law [9,28,29]). This skewed distribution has several hierarchies called circles. The sizes of these circles (the number of social relationships on the inside of each circle) are 5, 15, 50, 150, 500 and 1500, respectively [26,30]. That is, the ratios between neighbouring circles of social relationships are roughly three irrespective of social grooming methods, e.g. face to face, phone, Facebook and Twitter [30].

I aim to explore how and why humans use various social grooming methods and how those methods affect human behaviour and social relationship forms. For this purpose, I analyse the strength of the trade-off between the number and mean strength of social relationships as a key feature of social grooming methods. For this analysis, I extend the model [9] for *C* = *Nm*^*a*^ from *a* > 1 to *a* > 0 by introducing individuals' strategies about the amount of social grooming behaviour. This model explains not only online communication but also offline communication. The key feature restricts social relationship forms (size and distributions of strengths). Therefore, humans should change social grooming strategies depending on the trade-off, i.e. they tend to use several social grooming methods for constructing various strengths of social relationships. My model is supported by the common features of 13 diverse communication datasets including primitive human communication (face-to-face and communication in a small community constructed by kin and friends), modern communication (phone calls, e-mail, SNS and communication between unrelated people) and non-human primate communication (Chacma baboons). The model connects social behaviour and social relationship forms with a trade-off between the number and mean strength of social relationships.

## 2. Data analysis

**Figure 1.**
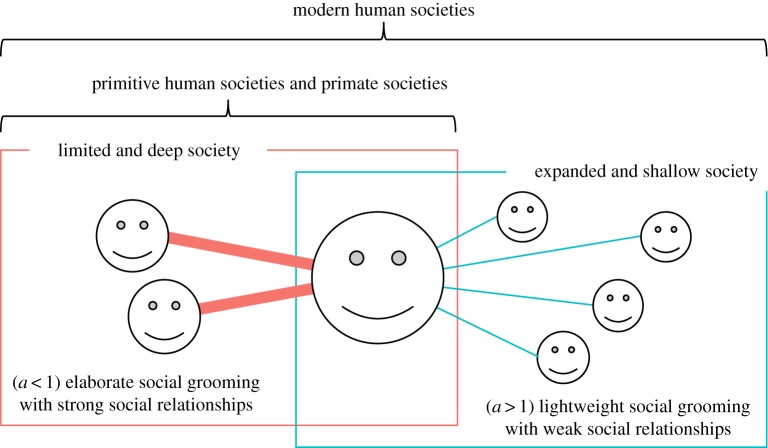
I found two types of social grooming (elaborate social grooming (orange) and lightweight social grooming (green)) and social relationship forms depending on them. People and non-human primates tended to do elaborate social grooming (e.g. face-to-face communication and fur cleaning in primates) with close social relationships. This social grooming generated limited and deep societies. On the other hand, people tended to do lightweight social grooming (e.g. SNS and e-mail) with weak social relationships. This social grooming generated expanded and shallow societies.

I found two types of social grooming methods based on the trade-off between the number and strength of social relationships (figure 1). One was ‘elaborate social grooming’, which was face to face and by phone (face to face (Pachur) [31]^1^ and phone (Pachur) [31] (see footnote 1)), in kin and friends (mobile phone (friends and family) [32]^2^ and short message service (SMS (friends and family)) [32] (see footnote 2)) and Chacma baboon social grooming (baboon group A and B) [6].^3^ This should be nearer to primitive human communications than the others. That is, these communications tend to bind individuals due to time and distance constraints or in primitive groups constructed by kin and friends. Another one was ‘lightweight social grooming’ which was by SNS and e-mail (Twitter [33],^4^ 755 group chat [9],^5^ 755 wall communication [9] (see footnote 5), Ameba Pigg [9] (see footnote 5) and e-mail/letter (Pachur) [31] (see footnote 1)) and in relationships between unrelated people (mobile phone (dormitory) [34]^6^ and SMS (dormitory) [34] (see footnote 6)), which has appeared in the modern age. These communications tend to unbind humans from time and distance constraints. This tended to be used with unrelated people. Details of datasets noted in brackets are shown in the Datasets section in the electronic supplementary material.

**Figure 2.**
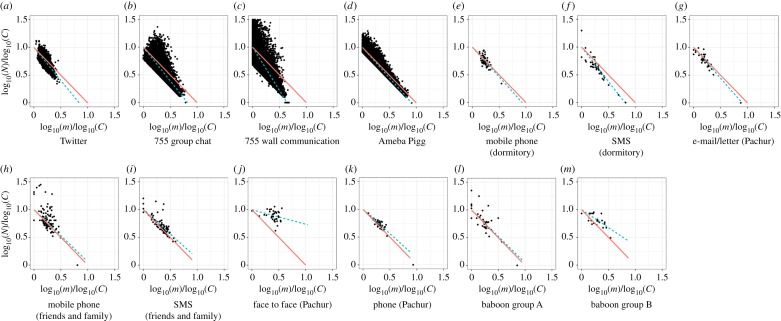
Two types of social relationships separated by the trade-off relationships between *N* and *m* (panels *a*–*g* are adapted from [9]). This figure shows log*N*/log*C* and log*m*/log*C* to remove the effect of covariate *C* from the relationships between *N* and *m*. These were separated by parameter *a* on *C* = *Nm*^*a*^ model (see table 1 for details). (*a*–*g*) Lightweight social grooming (*a* > 1) and (*h*–*m*) elaborate social grooming (*a* < 1). The black points show user behaviour data, orange lines show the regression lines of the models when *a* = 1 (*C* = *Nm*), and green dashed lines show the regression lines of *C* = *Nm*^*a*^ models. *a* < 1 shows a weak trade-off between *N* and *m*, as a result, people tended to construct a few strong social relationships. On the other hand, *a* > 1 shows a strong trade-off between *N* and *m*, as a result, people tended to construct many weak social relationships.

**Figure 3.**
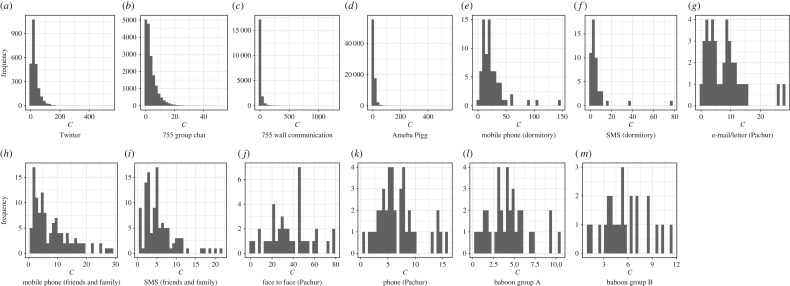
(*a*–*m*) Distributions of total social grooming cost *C*. There is a difference of how frequently individuals used each social grooming method.

Both were divided by parameter *a* on *C*_*i*_ = *N*_*i*_*m*^*a*^_*i*_ model (figure 2), where *a* showed strengths of the trade-off between *N*_*i*_ and *m*_*i*_, and individual *i*'s total social grooming cost was *C*_*i*_, *N*_*i*_ was *i*'s number of social relationships, *m*_*i*_ was *i*'s mean strength of their social relationships ($m_{i}=\sum_{j=1}^{N_{i}}d_{ij}/N_{i}$), and *d*_*ij*_ was the total number of the days on which *i* did social grooming to individual *j* (the strength of social relationships between *i* and *j*). *C*_*i*_ represents *i*'s available social capital on each social grooming method which varied widely among individuals (figure 3). I estimated statistically parameter *a* of the datasets by using a regression model: log*N*∼Normal( − *a*log*m* + *b*log*u*, *σ*), where *u* was the number of days of participation for each person and *σ* was a standard deviation. That is, this model assumed that a user's total social grooming costs were equal to the *b*th power of the number of days for which they had participated in the activity (*C* = *u*^*b*^) [9]. *u*^*b*^ was entered as a covariate to control the usage frequency of social grooming methods. Table 1 shows the details of this regression result. A few *p*-values of *a* were larger than 0.05, but social grooming methods overall seem to be divided by parameter *a*.

**Table 1.** The results of the regression analysis in figure 2. The *t*-values and the *p*-values of *a* measuring the statistical uncertainty in coefficient *a* are larger than 1 when *a* > 1 and the statistical uncertainties in coefficient *a* are smaller than 1 when *a* < 1. The *t*-values and the *p*-values of *b* measuring the statistical uncertainty in coefficient *b* are not equal to 0. The coefficient *a* was larger than 1 in lightweight social grooming methods (Twitter, 755 group chat, 755 wall communication, Ameba Pigg, mobile phone (dormitory), SMS (dormitory), and e-mail/letter (Pachur)). On the other hand, the coefficient *a* was smaller than 1 in elaborate social grooming methods (mobile phone (friends and family), SMS (friends and family), face to face (Pachur), phone (Pachur), baboon group A and baboon group B). Their adjusted *R*-squared values were very high.

social grooming method	adjusted *R*^2^	coef.	estimate	s.e.	*t*-value	*p*-value
Twitter	0.990	*a*	1.18957	0.02326	51.15	<4.4 × 10^−16^
		*b*	1.30935	0.00682	192.12	<2.0 × 10^−16^
755 group chat	0.974	*a*	1.21423	0.00464	46.17	<2.0 × 10^−16^
		*b*	1.26977	0.00229	553.5	<2.0 × 10^−16^
755 wall	0.959	*a*	1.56214	0.00625	89.94	<2.0 × 10^−16^
communication		*b*	1.47639	0.00277	533.2	<2.0 × 10^−16^
Ameba Pigg	0.997	*a*	1.09541	0.00074	128.24	<2.0 × 10^−16^
		*b*	1.09395	0.00031	3487	<2.0 × 10^−16^
mobile phone	0.994	*a*	1.07332	0.15756	0.48	<3.2 × 10^−1^
(dormitory)		*b*	1.25628	0.04689	26.795	<2.0 × 10^−16^
SMS	0.990	*a*	1.24089	0.07815	3.08	<3.5 × 10^−3^
(dormitory)		*b*	1.21949	0.02995	40.72	<2.0 × 10^−16^
e-mail/letter	0.988	*a*	1.10884	0.17744	0.613	<2.7 × 10^−1^
(Pachur)		*b*	1.14310	0.04267	26.789	<2.0 × 10^−16^
mobile phone	0.978	*a*	0.93982	0.13896	0.43	<3.3 × 10^−1^
(friends and family)		*b*	1.23703	0.04531	27.300	<2.0 × 10^−16^
SMS	0.992	*a*	0.87749	0.05906	2.07	<2.0 × 10^−2^
(friends and family)		*b*	1.04883	0.02360	44.45	<2.0 × 10^−16^
face to face	0.990	*a*	0.26334	0.18489	3.984	<1.6 × 10^−4^
(Pachur)		*b*	1.02148	0.08065	12.666	<5.1 2.0 × 10^−15^
phone	0.997	*a*	0.87585	0.09219	1.35	<9.3 × 10^−2^
(Pachur)		*b*	1.07172	0.03087	34.717	<2.0 × 10^−16^
baboon group A	0.985	*a*	0.95319	0.16301	0.29	<3.9 × 10^−1^
		*b*	1.17293	0.05034	23.30	<2.0 × 10^−16^
baboon group B	0.991	*a*	0.64714	0.13587	2.60	<8.2 × 10^−3^
		*b*	1.07354	0.04763	22.539	<2.0 × 10^−16^

Here *a*≠1 suggests that social grooming behaviour depends on the strength of social relationships because *a* will be 1 when social grooming behaviour does not relate to the strength of social relationships ($C_{i}=N_{i}m_{i}=\sum_{j=1}^{N_{i}}d_{ij}$). Here *a* < 1 shows that people have stronger social relationships than when *a* = 1 because the effect of strong relationships (large *m*) to cost *C* is smaller than when *a* = 1 ($C_{i}=N_{i}m_{i}^{a}<\sum_{j=1}^{N_{i}}d_{ij}$ when *m* > 1). By contrast, *a* > 1 shows that people have weaker social relationships than when *a* = 1 because of the effect of strong relationships (large *m*) to cost *C* is larger than when *a* = 1 ($C_{i}=N_{i}m_{i}^{a}>\sum_{j=1}^{N_{i}}d_{ij}$ when *m* > 1). I call the social grooming methods of *a* > 1 (i.e. modern methods) lightweight social grooming (figure 2*a*–*g*) and the methods of *a* < 1 (i.e. primitive methods) elaborate social grooming (figure 2*h*–*m*). That is, the trade-off of elaborate social grooming between the number and strength of social relationships is smaller than that of lightweight social grooming. On the other hand, the time and effort of lightweight methods are less than those of elaborate methods [35].

This trade-off parameter *a* affected human social grooming behaviour. People invested more time in their closer social relationships, i.e. the amount of social grooming between individuals increased with their strength of social relationships. Additionally, *a* changed people's trends of social relationship constructions. People having limited and deep social relationships tended to do frequent social grooming (amount of social grooming was large) when *a* < 1. On the other hand, people having expanded and shallow social relationships tended to do frequent social grooming when *a* > 1. I show these in the following.

**Figure 4.**
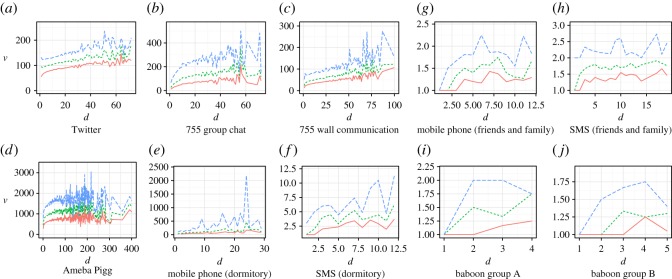
Increasing amount of social grooming per day *v* by strengths of social relationships *d* (panels *a*–*h* are adapted from [9]). The definitions of an amount of social grooming are shown in electronic supplementary material, table S2. The orange lines are the 25th percentile, the green dotted lines are the 50th percentile and the blue dashed lines are the 75th percentile. These are shown for cases where the number of samples was more than 20 (the ranges of *d* of (*e*–*j*) are short because these were smaller datasets).

**Figure 5.**
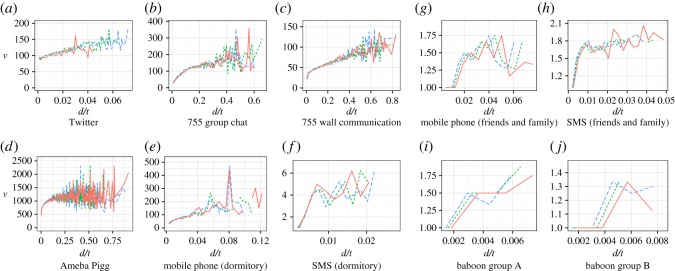
The gradients of the amount of social grooming depending on social grooming density as distinct from those depending on social grooming frequency (panels *a*–*h* are adapted from [9]). This figure shows a comparison of the medians of *v* for each social grooming density (*d*/*t*) for different periods (*t* is the number of elapsed days). Each line represents entire periods (orange lines), nine-tenths of the periods (green dotted lines) and eight-tenths of the periods (blue dashed lines). These are shown when the number of samples is more than 20 (the ranges of *d* of (*e*–*j*) are short because these were smaller datasets).

Figures 4 and 5 and the previous work [9] show that the amount of social grooming from individual *i* to individual *j* tended to increase with the density of social grooming *w*_*ij*_, where *w*_*ij*_ = *d*_*ij*_/*t* and *t* was the number of elapsed days from the start of observation, i.e. the amount of social grooming did not depend on *t*. I modelled this phenomenon as linear increase which was the simplest assumption, that is, *v*(*w*_*ij*_) = *αw*_*ij*_ + 1, where *v*(*w*_*ij*_) was the amount of social grooming from individual *i* to individual *j* and *α* was a parameter.

This assumption and the definition of *C*_*i*_ = *N*_*i*_*m*^*a*^_*i*_ suggest a relationship between individual social relationship trends and their total amount of social grooming. Here *m*_*i*_ shows individual *i*'s sociality trend (mean limitation and depth of its social relationships) by the trade-off between *N* and *m*. The total amount of social grooming per day to reinforce social relationships is

2.1 $$G(a,\alpha ;C,m)=\frac{\alpha C(m^{1-a}-m^{-a})}{T},$$

where *T* is the number of days of the data periods. $m_{i}=N_{i}^{-1}\sum_{j=1}^{N_{i}}d_{ij}(T)=TN_{i}^{-1}\sum_{j=1}^{N_{i}}w_{ij}$ is based on the definition of *m* and *w*. Therefore, the total amount of social grooming *V*_*i*_ is $\sum_{j}^{N_{i}}v(w_{ij})=\alpha m_{i}N_{i}T^{-1}+N_{i}$. *G*(*a*, *α*;*C*, *m*) is acquired by subtracting the total cost for creating social relationships (*V*_0_ = *α* + *T*) from *V*_*i*_ (see the Development of equation 1 section in electronic supplementary material). *α* was decided by the following simulation where an individual-based model was fitted to the datasets. This equation shows that people who have large *m* (i.e. limited and deep social relationships) have a large amount of social grooming when doing elaborate methods (*a* < 1; the orange line in figure 13*d*). On the other hand, people who have small *m* (i.e. expanded and shallow social relationships) have a large amount of social grooming when doing lightweight methods (*a* > 1; the green line in figure 13*d*). That is, *G*(*a*, *α*;*C*, *m*) shows that social grooming methods were used depending on the strength of social relationships (elaborate social grooming used for strong social relationships and lightweight social grooming used for weak social relationships). The threshold of both social grooming methods was *a* = 1. The amount of social grooming for construction of new social relationships *G*_0_ does not depend on *a* (*G*_0_≃*N*; see Development of equation 1 section in electronic supplementary material for details).

**Figure 6.**
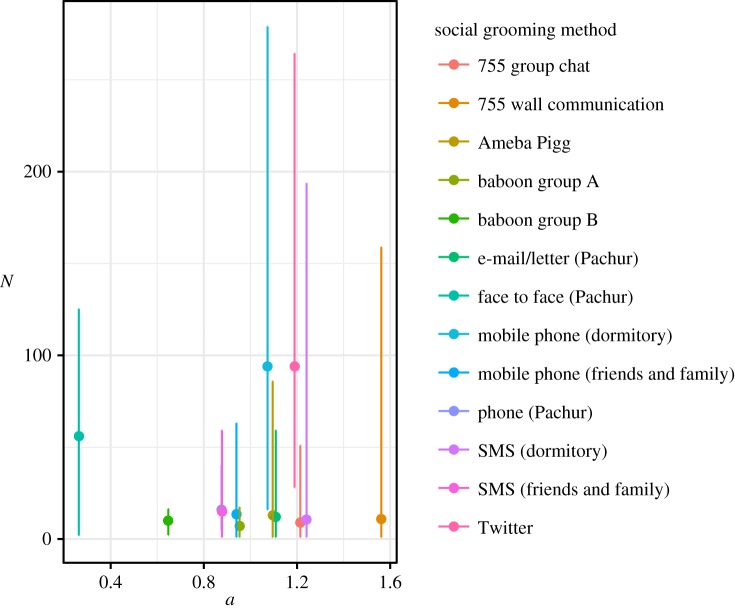
The number of social relationships for each social grooming method. Points show the medians of the number of social relationships. Error bars show ranges from 2.5 percentile to 97.5 percentile. The number of social relationships for each social grooming method did not show clear trends between *a* and the number of social relationships. This seemed to be caused by the difference in data gathering on the datasets.

Here *a* changed the peak of a total amount of social grooming *G* depending on sociality trend *m*; nevertheless, the number of social relationships for each social grooming method did not show clear relationships to *a* (figure 6). Additionally, their number of social relationships was smaller than the general number (about 150 [36,37]). There may be the problem for evaluating the number of social relationships due to the differences in data gathering on the datasets. There may not be a difference of the number of social relationships among the datasets, because the previous studies [36,37] showed that the number of social relationships did not particularly depend on social grooming methods.

**Figure 7.**
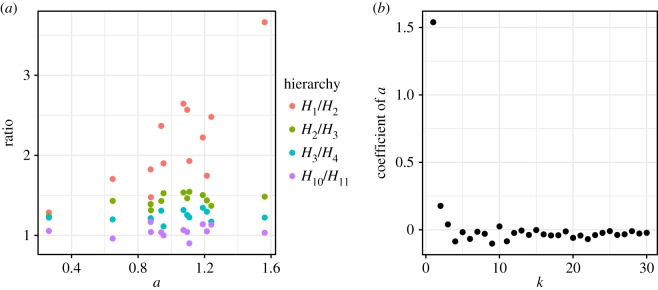
Relationships between *a* and the size ratio between neighbouring hierarchies of social relationships (*H*_*k*_/*H*_*k*+1_), where *H*_*k*_ is the number of social relationships when *d*≥*k* (i.e. smaller *k* shows the outer hierarchies). (*a*) Examples of the size ratios (*k* = 1, 2, 3 and 10). (*b*) Coefficient of *a* in a regression model (*β*_1_ in *H*_*k*_/*H*_*k*+1_∼ Normal(*β*_1_*a* + *β*_0_, *α*)) on each *k* (see the electronic supplementary material, table S1 for details), where the *p*-values of coefficients of *a* of *k* = 1, 2 were significant (at the 5% level). That is, these ratios increased with *a* only when *k* is small. This suggests that the trade-off parameter *a* mainly affected very weak social relationships. (*a*) Ratios between neighbouring hierarchies of social relationships. (*b*) Coefficients of *a* in the ratio.

Here *a* affected the ratio of very weak social relationships. Figure 7 shows the size ratio between neighbouring hierarchies of social relationships, i.e. *H*_*k*_/*H*_*k*+1_, where *H*_*k*_ is the number of social relationships when *d*≥*k*. That is, *H*_1_ is the number of all social relationships, *H*_2_ is the number of social relationships excluding relationship *d* = 1, and *H*_*k*_ with large *k* is the number of close relationships. The trade-off parameter *a* only affected *H*_*k*_/*H*_*k*+1_ when *k* was very small. Thus, the strength of trade-off *a* related only to very weak social relationships which seemed to be the circle of acquaintances [30].

## 3. Individual-based simulations

### 3.1. Model

In the previous section, I found the threshold *a* = 1 on social grooming behaviour (equation (2.1)). By contrast, a threshold was not observed on social relationship forms due to the differences of data gathering and sampling on the datasets. In this section, I conducted individual-based simulations to analyse changes of social relationship forms depending on *a* under the same conditions.

I constructed an individual-based model to explore the effects of the trade-off parameter *a* on social relationship forms based on the monotonic increasing of *v*(*w*_*ij*_) and the difference of the peak of *G*(*a*, *α*;*C*_*i*_, *m*_*i*_) depending on *a* (equation (2.1)). That is, individual *i* does social grooming to individual *j*, the amount being proportional to *w*_*ij*_. *i*'s total amount of social grooming for reinforcing all social relationships is *G*(*a*, *α*;*C*_*i*_, *m*_*i*_). Additionally, I assumed the Yule–Simon process on social grooming partner selection, because people basically do act this way [9,31] (see also the electronic supplementary material, figure S2). In the Yule–Simon process, which is one of the generating processes of power-law distributions [38], individuals select social grooming partners in proportion to the strength of their social relationships, that is, the individuals reinforce their strong social relationships. In the model, individuals construct new social relationships and reinforce existing social relationships, where they pay their limited resources *R* = *G*(*a*, *α*;*C*_*i*_, *m*_*i*_) for the reinforcement. This model is an extension of the individual-based model of the previous study [9] in which *G* is introduced. The Source Code of the Individual-based Simulations section in electronic supplementary material shows a source code of this model.

I consider two types of individuals, groomers and groomees. Groomers construct and reinforce social relationships using their limited resources *R* (that is, time), based on these assumptions and the Yule–Simon process. I use the linear function *v*(*w*_*ij*_) = *αw*_*ij*_ + 1 as the amount of social grooming from groomer *i* to groomee *j* as with the above section.

I conducted the following simulation for *T* days to construct social relationships *d*_*ij*_ in simulation experiments 1 and 2. Individuals have a social relationship where strength is 1 as the initial state. On each day *t*∈[1, *T*], groomer *i* repeats the following two processes for its resource *R*_*i*_ > 0. *R*_*i*_ is reset to an initial value *G*(*a*, *α*;*C*_*i*_, *m*_*i*_) before each day *t*. Each *i* spends *R*_*i*_ reinforcing its social relationships.

#### 3.1.1. Creating new social relationships.

Each *i* creates social relationships with strangers (groomees). The strength of a new social relationship with *j* (*d*_*ij*_) is 1. The number of new relationships obeys a probability distribution *Poisson*(*p*_*i*_), where *p*_*i*_ is (*N*_*i*_ − 1)/*T*. Therefore, it is expected that *i* has *N*_*i*_ social relationships until day *T*, because the relationship between *N* and *a* was unclear in the previous section, the expected value of *N* does not depend on *a* and is constant. This setting should be natural because the previous studies [37,39] showed independence of *N* from social grooming methods. Creating new relationships does not spend *R*.

#### 3.1.2. Reinforcing existing social relationships.

Here *i* also reinforces its social relationships. Each *i* selects a social grooming partner *j* depending on a probability proportional to the strength of the social relationships between *i* and *j*, then *i* adds 1 to *d*_*ij*_ (that is, the Yule–Simon process) and spends the amount of social grooming *v*(*w*_*ij*_) from *R*_*i*_ (if *R*_*i*_ < *v*(*w*_*ij*_), then *i* adds *R*_*i*_/*v*(*w*_*ij*_) to *d*_*ij*_ and *R*_*i*_ becomes 0). Each *i* does not perform the act of social grooming more than once with the same groomees in each day *t*. Therefore, selected groomees are excluded from the selection process of a social grooming partner *j* on each day *t*.

### 3.2. Simulation experiment 1: checking the model consistency

In this experiment, I confirmed a consistency between *C* = *Nm*^*a*^ and two assumptions of social grooming behaviour (*G*(*a*, *α*;*C*_*i*_, *m*_*i*_) and *v*(*w*_*ij*_) = *αw*_*ij*_ + 1). Therefore, I fitted my individual-based model to the datasets optimized by unknown parameter *α*. I used actual values of the datasets as *a*, *T* and *C*_*i*_ in each simulation, where *a* was the values in figure 2 and table 1, *T* was the period for each dataset, and *C* was the 75th percentile of *u*^*b*^. *N*_*i*_ was equally divided in a logarithmic scale (*N*∈[1, *T*]). Unknown parameter *α* was calculated by the optimization which decreased error values of simulations ($e=\sum_{i=1}^{M}\{{\left(\log N_{i}-\log N_{i}^{\prime }\right)}^{2}+{\left(\log m_{i}-\log m_{i}^{\prime }\right)}^{2}\}/M$), where *m*_*i*_ = (*C*/*N*_*i*_)^(1/*a*)^, *M* was the number of individuals (*M* = 30), and *N*′_*i*_ and *m*′_*i*_ were calculated by simulation results (social relationship strengths *d* of each individual).

Next, I calculated social relationship forms (distributions of *d*_*ij*_) in each *a* by using actual settings (*T*, *C*_*i*_, *N*_*i*_) and the optimized *α*, where *T* was the period for each dataset, *C*_*i*_ was individual *i*'s *u*^*b*^_*i*_, and *N*_*i*_ was *i*'s number of social relationships in each dataset.

**Figure 8.**
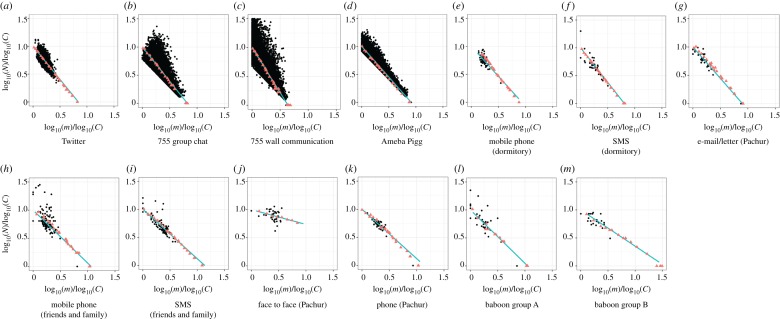
(*a*–*m*) The simulation model fit to the datasets (simulation experiment 1). This shows consistency of the model with two assumptions (*v*(*w*_*ij*_) = *αw*_*ij*_ + 1 and *G*(*a*, *α*;*C*, *m*)). The simulations made fit the results to the regression lines of all datasets (that is, green and dashed lines in figure 2), where these fitting parameters were *α*. Very good fits were observed between the simulation results (orange triangles) and the regression lines (green lines). The parameters *α* of *v*(*w*_*ij*_) and *G*(*a*, *α*;*C*, *m*) are shown in table 2.

**Figure 9.**
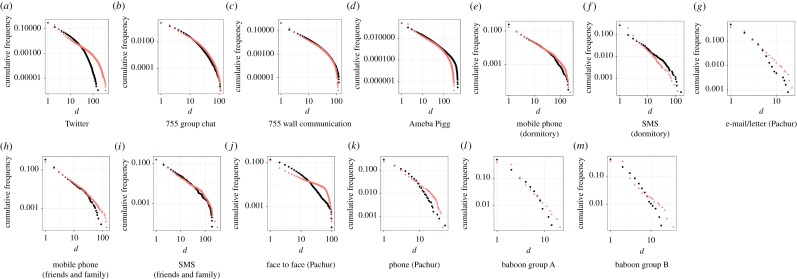
(*a*–*m*) The cumulative distributions of strengths of social relationships *d*_*ij*_ of each dataset (black points) and a simulation result of each dataset (orange triangle points) in simulation experiment 1. The simulation results roughly show similar trends with each dataset excluding face to face (Pachur).

This model fitted all datasets (figure 8 and table 2). Their distributions of social relationships *d*_*ij*_ were roughly similar to actual distributions excluding face to face (Pachur) (figure 9). Additionally, the amount of social grooming predicted by equation (2.1) with the optimized *α* showed a high correlation with the actual amount of social grooming in each dataset (table 3). That is, this model roughly has an explanation capacity for generating the process of social relationships, depending on human social grooming behaviour, regarding the trade-off constraint. The difference between the simulation result and face to face (Pachur) dataset may have been because the approximations of this model did not work with small *a*.

**Table 2.** The parameters *α* of *v*(*w*_*ij*_) and *G*(*a*, *α*;*C*, *m*) in experiment 1.

communication system	*α*
Twitter	1.034927
755 group chat	1.206970
755 wall communication	1.131248
Ameba Pigg	0.946045
mobile phone (dormitory)	2.734375
SMS (dormitory)	1.019287
e-mail/letter (Pachur)	1.708984
mobile phone (friends and family)	1.887512
SMS (friends and family)	1.562500
face to face (Pachur)	2.148438
phone (Pachur)	1.660156
baboon group A	1.044464
baboon group B	1.020813

**Table 3.** High correlations of log*G*(*a*, *α*;*C*_*i*_, *m*_*i*_) and log*G*_*i*_, where *G*_*i*_ is a summation of *i*'s amount of social grooming per day without initial social grooming with strangers, i.e. the actual amount of social grooming. *α* was decided by experiment 1.

communication system	correlation	*p*-value
Twitter	0.8399704	<2.0 × 10^−16^
755 group chat	0.7057249	<2.0 × 10^−16^
755 wall communication	0.8130153	<2.0 × 10^−16^
Ameba Pigg	0.7678780	<2.0 × 10^−16^
mobile phone (dormitory)	0.7434336	5.0 × 10^−14^
SMS (dormitory)	0.9079204	<2.0 × 10^−16^
mobile phone (friends and family)	0.9749272	<2.0 × 10^−16^
SMS (friends and family)	0.9733798	<2.0 × 10^−16^
baboon group A	0.9789251	<2.0 × 10^−16^
baboon group B	0.9790673	<2.0 × 10^−16^

### 3.3. Simulation experiment 2: effects of social grooming methods

In this experiment, I analysed the effect of parameter *a* on the structure of social relationships by using the model. First, I calculated error value *e*_*aα*_ in each *a* and *α*, where *a* was {0.50, 0.55, …, 2.00} and *α* was {1.00, 1.02, …, 3.00}. That is, the number of combinations is 31 × 101 = 3131. In each simulation, *T* was the period for the Twitter dataset, *C*_*i*_ was the 75th percentile of *u*^*b*^ in the Twitter dataset, *N*_*i*_ was equally divided in a logarithmic scale and *M* = 30. Each *e*_*aα*_ was calculated 50 times, i.e. there are 5050 results on each *a*. I used *α* which was ranked in the lowest 20 of *e*_*aα*_ of each *a*.

Next, I calculated social relationship forms (the distributions of social relationship strengths *d*_*ij*_) in each *a* by using actual settings (*T*, *C*_*i*_, *N*_*i*_) and *α*, where *T* was the period for the Twitter dataset, *C*_*i*_ was individual *i*'s *u*^*b*^_*i*_, *N*_*i*_ was *i*'s number of social relationships in the Twitter dataset, and *M* was the number of people in the Twitter dataset.

Firstly, I evaluated a power-law coefficient *ϕ* as overall effects of *a* on social relationship forms because social relationship forms (distributions of social relationship strengths *d*_*ij*_) follow power-law distributions [9,28,29]. Secondly, I analysed the ratio between neighbouring circles of weak social relationships which depended on *a* in the previous section (figure 7).

**Figure 10.**
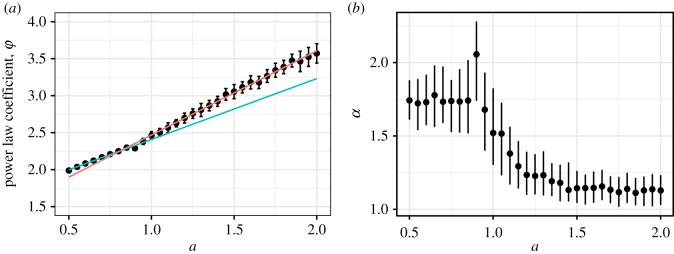
A change of skewed social relationship forms (power-law coefficients *φ* on distributions of social relationship strengths *d*_*ij*_) around a threshold *a* = 0.8 (*a*). The black points are the mean *φ* in the lowest 20 error value *e*_*aα*_ in each *a* and the error bars are standard deviations of these values. The orange and green lines are the result of the linear regression model with the threshold at *a* = 0.8. That is, the green line shows the gradient of coefficients *φ*(*β*_2_) in *a*∈[0.5, 0.8) and the orange line shows the gradient of *φ*(*β*_1_) in *a*∈[0.8, 2.0]. The gradient of *φ* when *a*≥0.8 was larger than when *a* < 0.8, i.e. expanded and shallow social relationship forms. That is, power law coefficients *φ* when *a*≥0.8 tended to change more than when *a* < 0.8. Simultaneously, *α* was dramatically decreased when *a*≥0.8, i.e. individuals decreased social grooming time for strong social relationships (*b*). The black points show mean *α* in the lowest twenty error value *e*_*aα*_ in each *a*. The error bars show their standard deviations. These values of the coefficients and *α* were calculated by the individual-based simulations. (*a*) The effect of *a* on power-law coefficient *φ* of social relationships strengths. (*b*) The effect of *a* on *α*.

I found that *a* changed the social relationship forms *ϕ* and social behaviour parameter *α* where the threshold was around *a* = 0.8 (figure 10) because a linear regression model with the threshold at *a* = 0.8 was more accurate than a model without the threshold, i.e. the former had smaller Akaike information criterion (AIC) than the latter. The former is *ϕ*∼ Normal(*β*_1_*af* + *β*_2_*a*(1 − *f*) + *β*_3_*f* + *β*_0_, *σ*), where *f* = 1 when *a*≥0.8, otherwise *f* = 0 and *σ* is standard deviation (AIC: −1452.6; table 4). The latter is *ϕ*∼Normal(*β*_1_*a* + *β*_0_, *σ*) (AIC: −1400.9). That is, the changes of *a* in *a* < 0.8 (*β*_2_) had a smaller effect on power-law coefficients *ϕ* of social relationship forms than in *a*≥0.8 (*β*_1_). This shows that strong social relationships decreased in *a*≥0.8 because individuals having expanded and shallow social relationships have more of the amount of social grooming than individuals having limited and deep social relationships (*G*(*a*, *α*;*C*, *m*)). This threshold seems to be due to the threshold of *G*(*a*, *α*;*C*, *m*). The difference between this threshold (*a* = 0.8) and the threshold of *G*(*a*, *α*;*C*_*i*_, *m*_*i*_) (*a* = 1) may have been because of the approximations of this model.

**Table 4.** The results of the linear regression model with the threshold at *a* = 0.8. This adjusted *R*^2^-value was 0.978.

coefficient	estimate	s.e.	*t*-value	*p*-value
*β*_1_	0.87282	0.07979	10.940	0<2.0 × 10^−16^
*β*_2_	0.27829	0.08032	3.465	<5.68 × 10^−15^
*β*_3_	-0.24724	0.05208	-4.747	<2.56 × 10^−6^
*β*_0_	1.55549	0.05033	30.906	0<2.0 × 10^−16^

Additionally, *α* in *a* < 0.8 was larger than *α* in *a*≥0.8 excluding *a* = 0.8. Interestingly, *α* also drastically changed in the range of 0.8 ≤ *a* ≤ 1.3. That is, individuals in *a*≥0.8 decreased the amount of social grooming *v*(*w*_*ij*_) with close social relationships as compared to *a* < 0.8.

**Figure 11.**
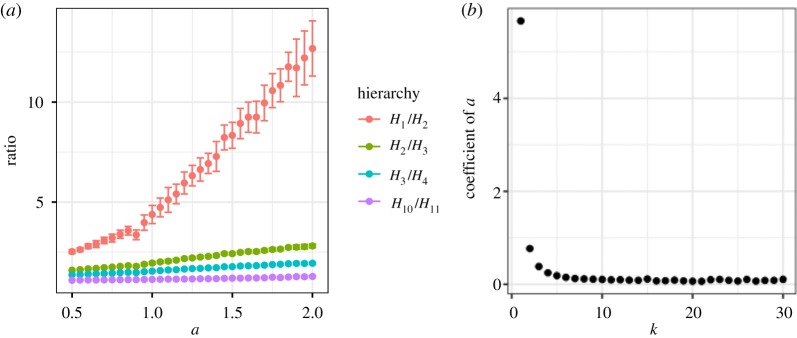
The ratio between neighbouring hierarchies (*H*_*k*_/*H*_*k*+1_). *H*_*k*_ is the number of social relationships when *d* > *k*. (*a*) Examples of the size ratios (*k* = 1, 2, 3, and 10). (*b*) Coefficients of *a* in a regression model (*β*_1_ in *H*_*k*_/*H*_*k*+1_∼ Normal(*β*_1_*a* + *β*_0_, *σ*)) on each *k* (see electronic supplementary material, table S2, for details), where all *p*-values of coefficients of *a* were significant (at the 5% level). These ratios increased with *a* only when *k* is very small. The ratio between neighbouring hierarchies of very weak social relationships *H*_1_/*H*_2_ significantly changed around *a* = 0.8.

**Figure 12.**
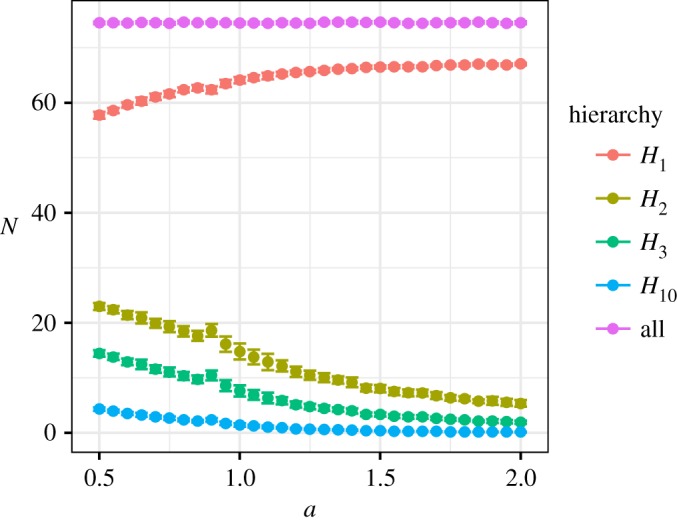
Each hierarchy size depends on *a*. Points show the means of the number of social relationships. Error bars show standard deviation. Hierarchy size *H*_1_ including very weak social relationships increased with *a*. By contrast, hierarchy sizes *H*_*k*_ for *k*≥2 decreased with *a*. The number of social relationships (all) was approximately constant because this was determined by the settings of the simulation.

Here *a* affected the ratio of very weak social relationships. Figure 11 shows the size ratio between neighbouring hierarchies of social relationships, i.e. *H*_*k*_/*H*_*k*+1_, where *H*_*k*_ is the number of social relationships when *d* > *k*. That is, *H*_1_ includes all social relationships excluding relationships of *d* ≤ 1 (one time interactions), *H*_2_ is the number of social relationships excluding relationship *d* ≤ 2, and *H*_*k*_ with large *k* is the number of close relationships. There is a difference of the definition between this *H* and *H* in the Data analysis section due to the difference of the definition between *d* in this section (positive real number) and *d* in the Data analysis section (natural number). The ratio between neighbouring hierarchies of very weak social relationships *H*_1_/*H*_2_ significantly changed around *a* = 0.8. On the other hand, *H*_*k*_/*H*_*k*+1_ for *k*≥2 gradually increased with *a*. This was due to the fact that *H*_1_ increased with the increase *a* and *H*_*k*_ when *k*≥2 decreased with the increase *a* (figure 12). Thus, very weak social relationship forms were especially affected by *a* compared with strong social relationships.

**Figure 13.**
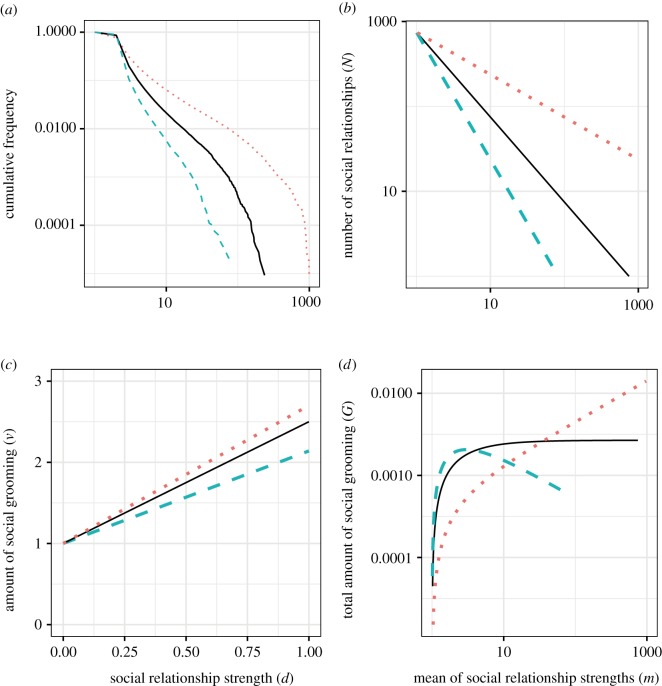
The relationship between social relationship forms (*a*), the trade-off of social grooming methods (*b*), and human social behaviour (*c* and *d*). In this figure, the orange lines show elaborate social grooming (*a* < 1), the green lines show lightweight social grooming (*a* > 1), and the black lines are the threshold (*a* = 1). Social grooming methods were separated by parameter *a* of trade-off relationships between the number and strength of social relationships (*b*). People changed their social grooming behaviour depending on *a*. The first was the gradients of an amount of social grooming from an individual to another (the gradients of (*c*)) increased with each strength of the social relationship. The stronger the social relationships, the greater the amount of social grooming was spent in those relationships. The gradients of lightweight social grooming (*a* > 1) were lower than those of elaborate social grooming (*a* < 1). The second was the total amount of social grooming to reinforce social relationships *G* of each individual (*d*). People having a few close social relationships tended to do social grooming frequently when *a* < 1 (the amount of social grooming was large). By contrast, people having many weak social relationships tended to do social grooming frequently when *a* > 1. The total amount of social grooming is normalized to compare each *a*. These two different behaviours depending on the trade-off changed social relationship forms (panel (*a*)). This figure was drawn by using the Twitter dataset with *a* = 0.5 (orange), *a* = 1.0 (black) and *a* = 1.5 (green) in simulation experiment 2. The total amount of social grooming in (*d*) is normalized to compare each *a*. (*a*) Social relationship forms depending on social grooming types, (*b*) trade-off between strength and number of social relationships, (*c*) increases of social grooming time depending on social relationship strengths and (*d*) total amount of social grooming depending on individuals' social relationship trends.

As a result, the social relationship forms were expanded and shallow in *a*≥0.8. This suggests that societies with lightweight social grooming had different properties when compared to societies with elaborate social grooming.

## 4. Discussion

I constructed a model of social relationship forms depending on human behaviour restricted by a trade-off between the number and strength of social relationships depending on social grooming methods. This model was supported by common features of 13 diverse communication datasets including primitive human communication, modern communication tools and non-human primates. By analysing the model, I found two types of social grooming (elaborate social grooming and lightweight social grooming). They made different social relationship forms. This was caused by people's social grooming behaviour depending on the different trade-off between the number and strength of social relationships. Both methods were separated by trade-off parameter *a* on *C* = *Nm*^*a*^ model (*a* < 1: elaborate social grooming; *a* > 1: lightweight social grooming). This separation was due to the total amount of social grooming *G* having the threshold *a* = 1. The model from the previous study [9] was expanded by adding *G*.

People tended to use elaborate social grooming in face-to-face communication and communication in small communities made up of kin and friends, i.e. the communities should be near primitive groups. Additionally, Chacma baboons also showed a similar trend. They tended to use this social grooming to reinforce close social relationships. That is, elaborate social grooming is a primitive method (i.e. *a priori*). This may be used in non-human primates, primitive human societies and close relationships of modern humans, i.e. these may not have a qualitative difference.

On the other hand, people tended to use lightweight social grooming in SNS, e-mail and communication in communities made up of unrelated people, i.e. the communities should be non-primitive groups. That is, this social grooming is posterior. People tended to use these methods to construct many weak social relationships. As a result, social relationship forms may have changed significantly when people have used lightweight social grooming. Therefore, human societies have become expanded and shallow. Figure 13 shows the relationship between the trade-off parameter of social grooming methods, human social behaviour and social relationship forms.

Owing to these differences, people use both social grooming methods depending on the strengths of social relationships [10,40,41] (electronic supplementary material, figure S1). A typical person who has various strengths of social relationships [26,30] uses elaborate social grooming (*a* < 1) for constructing a few close relationships and lightweight social grooming for having many weak social relationships. This is caused by the change of the peak of the function *G* around threshold *a* = 1.

The function *G* represents the total amount of social grooming for all relationships depending on sociality trend *m* and trade-off parameter *a* of social grooming. That is, individuals' total amount of social grooming are limited, and these limits (*G*(*a*, *α*;*C*, *m*)) depend on *m* and *a*. Thus, some individuals opt for lightweight social grooming (*a* > 1) a consequence of the fact that they want to have a larger network (small *m* and large *N*). In other words, two types of social grooming have emerged in consequence of social grooming strategies, which are how individuals distribute their limited resources by using several social grooming methods depending on trade-off parameter *a* of social grooming.

This qualitative difference between the two types of social grooming may be caused by the number of very weak social relationships an individual wants to create. The most affected relationships according to the difference of social grooming methods were the ratio between a hierarchy of social relationships including very weak social relationships and its neighbouring hierarchy. On elaborate social grooming, the trade-off parameter *a* has an insignificant effect on this ratio. By contrast, on lightweight social grooming, an increase of the trade-off parameter *a* increases the ratio. Humans may have acquired lightweight social grooming by the necessity to create many very weak social relationships (acquaintances). It seems to have been due to the increase in the number of accessible others or community sizes.

Some online social grooming methods (mobile phone (friends and family), SMS (friends and family), and phone (Pachur)) showed *a* < 1. These might have been caused by constructing non-weak social relationships. That is, the subjects of mobile phone (friends and family) and SMS (friends and family) were members of a young family living in a residential community which was constructed by kin and neighbours. Phone (Pachur) was used for communication closer relationships than e-mails when people use phone and e-mails (electronic supplementary material, figure S1). This could be achieved by comparing diverse communication datasets gathered by similar conditions.

Both social grooming methods also differ from a cost and effect perspective. The trade-off of interacting in close social relationships using elaborate methods is weaker than that of lightweight methods. On the other hand, the time and effort of lightweight methods are less than those of elaborate methods [35]. Social grooming with time and effort (elaborate social grooming) is effective to construct close social relationships [12,13,35]. Therefore, elaborate methods are suited to maintain a few close relationships. In addition, lightweight methods make it easier for people to have many weak social relationships [24,42].

The two types of social grooming methods have different roles. The role of elaborate methods should be to get cooperation from others. Humans tend to cooperate in close friends [8,20,21,37,43,44] because cooperators cannot cooperate with everyone [44,45]. The role of lightweight social grooming should be to get information from others. Weak social relationships tend to provide novel information [3,22,24].

Thus, it should be effective for people to use elaborate social grooming for close relationships while expecting cooperation from these relationships. They use widely lightweight social grooming in weak relationships while expecting novel information. As a result, the number of close relationships before and after SNS has not changed much [37]. Weak relationships after the appearance of SNS have been maintained effectively [9,10].

An advantage of having information would have increased with the changes of societies. As a result, lightweight social grooming has been necessary, and humans have had expanded and shallow social relationship forms. Humans probably have acquired this social grooming in the immediate past. This consideration will become clearer by analysing various datasets, e.g. other non-human primates, social relationship forms in various times and cultures, and other communication systems.

The way of using both methods may also depend on people's extroversion/introversion. In general, introverts have limited deep social relationships and extroverts have expanded shallow social relationships [46,47]. The diversity of the amount of social grooming *C* for each social grooming method suggests that usage strategies of the two types of social grooming methods differ for each person, e.g. introverts tend to do elaborate social grooming; in contrast, extroverts tend to do lightweight social grooming.

Primitive humans also used lower-cost social grooming methods (e.g. gaze grooming [1,2] and gossip [3]) than fur cleaning in non-human primates. These methods have evolved more for larger groups than that of non-human primates because these grooming methods enable humans to have several social relationships and require less time and effort [4,48]. However, my model does not distinguish these social grooming methods from social grooming in non-human primates; nevertheless, the model separates modern social grooming from primitive human social grooming. This may suggest that an appearance of lightweight social grooming significantly affects human societies nearly as much as the changes between non-human primates and primitive humans.

## Supplementary Material

ESM Fig. 1

## Supplementary Material

ESM Fig. 2

## Supplementary Material

ESM Section 1

## Supplementary Material

ESM Section 2

## Supplementary Material

ESM Section 3

## Supplementary Material

ESM Table 2

## Supplementary Material

ESM Table 2

## Notes

1 See https://doi.org/10.5061/dryad.pc54g.

2 See http://realitycommons.media.mit.edu/realitymining4.html.

3 See https://doi.org/10.5061/dryad.n4k6p/1.

4 See https://archive.org/details/twitter_cikm_2010.

5 See https://doi.org/10.6084/m9.figshare.3395956.v3.

6 See http://realitycommons.media.mit.edu/socialevolution.html.

## Data accessibility

All data needed to evaluate the conclusions in the paper are present in the paper and the electronic supplementary materials.
