# Supplementary material for: Two types of social grooming methods depending on the trade-off between the number and strength of social relationships
Source: R Soc Open Sci. 2018 Aug 1;5(8):180148. doi: 10.1098/rsos.180148 (PMC6124085; doi:10.1098/rsos.180148)
Supplement: ESM Fig. 1 [file rsos180148supp1.pdf]

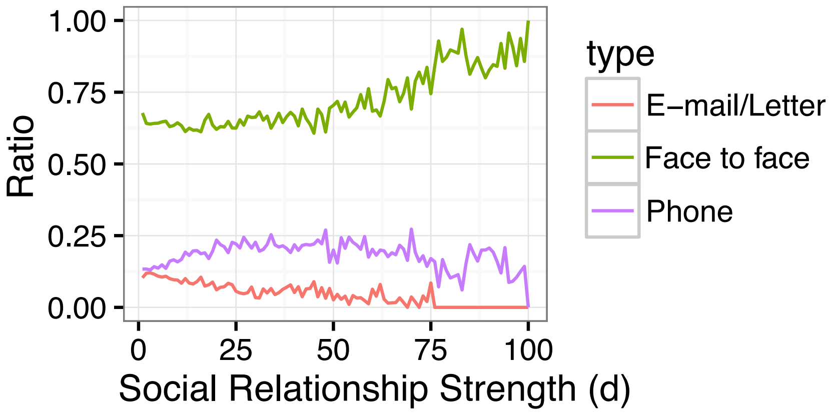

ESM Fig. 1: People tended to use more elaborate social grooming with stronger social relationships. This figure shows the ratio of social grooming methods on each strength of social relationships d. This figure was drawn by the author from Pachur et al. data-set [31]. The ratio of the lightweight method (E-mail/Letter) decreased with the increase of d. On the other hand, the ratio of the elaborate method (Face to face) increased with d. The ratio of Phone showed an intermediate tendency between both.
