# Supplementary material for: Two types of social grooming methods depending on the trade-off between the number and strength of social relationships
Source: R Soc Open Sci. 2018 Aug 1;5(8):180148. doi: 10.1098/rsos.180148 (PMC6124085; doi:10.1098/rsos.180148)
Supplement: ESM Fig. 2 [file rsos180148supp2.pdf]

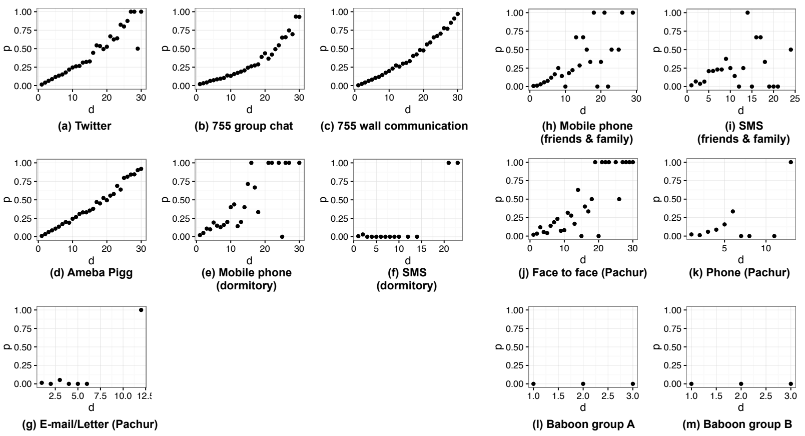

ESM Fig. 2: The Yule-Simon process on social grooming strategies (Fig. a-g are adapted from [9]). These figures show probability p of social grooming on days after each strength of social relationship d. The data periods were from the first thirty days. This indicates that the power law distributions were generated by the Yule-Simon process because the p of Fig. a-d was proportional to d, and these strategies subsequently generated the power law distributions. We did not observe the similar trends in the other data-sets due to insufficient data. However, Fig. e-k also show increasing trends. Therefore, they seem to obey the same process, because the generated distributions of all data-sets show the same distributions, i.e. power law distributions (black points in Fig. 9). These results were similar in a previous study [31].
