# Supplementary material for: Two types of social grooming methods depending on the trade-off between the number and strength of social relationships
Source: R Soc Open Sci. 2018 Aug 1;5(8):180148. doi: 10.1098/rsos.180148 (PMC6124085; doi:10.1098/rsos.180148)
Supplement: ESM Section 1 [file rsos180148supp3.pdf]

# 1 Data-Sets

I used thirteen diverse data-sets (see Table 1 for details): a) Twitter data (used as test set in the paper (Cheng et al., 2010)) recording interactions among $2,585$ people with $278,475$ relationships where an act of social grooming was defined as using the “mention” or “reply” functions to communicate with others. I used the number of characters per day as the amount of social grooming $v$; b) and c) Data from the Japanese SNS 755 (Takano and Fukuda, 2017) which provides two types of communication systems data which I treated as two different sets (see Fig. 1 of supplementary information in the paper (Takano and Fukuda, 2017) for specifications), namely data from group chats and that from wall communications. The former data records interactions among $17,796$ users with $238,611$ relationships, where I defined an act of social grooming as communicating in a chat limited to two members. The latter data records interactions among $20,000$ users with $534,475$ relationships, where I defined an act of social grooming as posting a comment on another’s wall. I removed data relevant to official users from both data-sets. I used the number of characters per day as the amount of social grooming $v$; d) Data from Japanese avatar chat Ameba Pigg [18] which records interactions among $76,379$ users with $1,610,710$ relationships (see Fig. 2 of supplementary information in the paper (Takano and Fukuda, 2017) for specifications) where I defined an act of social grooming as communicating in a chat limited to two members. I used the number of characters per day as the amount of social grooming $v$; e) Data from mobile phone calls (Madan et al., 2012) (Mobile phone (dormitory)), recording mobile phone calls among 73 people with $7,805$ relationships where I defined an act of social grooming as one call to another. The subjects were undergraduates in a dormitory who probably communicated with unrelated people (the same is true of item f). I used duration per day as the amount of social grooming $v$; f) Data from SMS (Madan et al., 2012) (SMS (dormitory)), which records SMS among 61 people with $2,266$ relationships where I defined an act of social grooming as sending one message to another. I used frequency of messaging per day as the amount of social grooming $v$ because I did not consider any information regarding the number of characters in this data-set; g) Data from E-mails and letters (Pachur et al., 2014) (E-mail/Letter (Pachur)), which records E-mail and letter communications among 40 people’s data for 100 days. This data was recorded together with data-sets of item j and k. These data-sets do not have the information of the amount of social grooming; h) Data from mobile phone calls (Aharony et al., 2011) (Mobile phone (friends & family)), recording mobile phone calls among 114 people with $2,264$ relationships where I defined an act of social grooming as a call to another. The subjects were members of a young family living in a residential community which was constructed by kin and neighbors (the same is true of item i). I used frequency of calls per day as the amount of social grooming $v$; i) Data from SMS (Aharony et al., 2011) (SMS (friends & family)), recording mobile phone calls among 109 people with $2,294$ relationships where I defined an act of social grooming as a call to another. I used frequency of messaging per day as the amount of social grooming $v$; j)

Data of face to face communication (Pachur et al., 2014) (Face to face (Pachur); see item g for details); k) Data from phone calls (Pachur et al., 2014) (Phone (Pachur); see item g for details); l) and m) Data of social grooming among Chacma baboons in two groups (Sick et al., 2014) (Baboon group A, B), which records fur cleaning behavior among 35 (B: 24) individuals with 266 (B: 225) relationships in 681 (B: 657) sessions, where the session is a quarter day (before 9 o'clock, 9-12 o'clock, 12-15 o'clock, and after 15 o'clock). I used the session instead of a day because this data-set does not have the date information. I used frequency of fur cleaning per session as the amount of social grooming $v$. Table 2 show the definitions of communication volume $v$.

In the data-sets from Twitter, 755 (group chat and wall communication) and Ameba Pigg, I limited the targets of analysis to active users who had greater number of social grooming days than the 50th percentile among Twitter users and the 75th percentile among 755 and Ameba Pigg users because these Internet service data-sets included many inactive users. In this paper, I defined the strength of social relationships $d_{ij}$ as the days on which individual $i$ does social grooming to individual $j$.

# References


Aharony, N., W. Pan, C. Ip, I. Khayal, and A. Pentland
2011. Social fMRI: Investigating and shaping social mechanisms in the real world. *Pervasive and Mobile Computing*, 7(6):643–659.

Cheng, Z., J. Caverlee, and K. Lee
2010. You are where you tweet: a content-based approach to geo-locating twitter users. In *Proceedings of the 19th ACM international conference on Information and knowledge management (CIKM '10)*, P. 759, New York, New York, USA. ACM Press.

Madan, A., M. Cebrian, S. Moturu, K. Farrahi, and A. S. Pentland
2012. Sensing the "health state" of a community. *IEEE Pervasive Computing*, 11(4):36–45.

Pachur, T., L. J. Schooler, and J. R. Stevens
2014. We'll meet again: Revealing distributional and temporal patterns of social contact. *PLoS ONE*, 9(1):e86081.

Sick, C., A. J. Carter, H. H. Marshall, L. A. Knapp, T. Dabelsteen, and G. Cowlishaw
2014. Evidence for varying social strategies across the day in Chacma baboons. *Biology letters*, 10:20140249.

Takano, M. and I. Fukuda
2017. Limitations of time resources in human relationships determine social structures. *Palgrave Communications*, 3:17014.

Table 1: Summaries of data-sets. $N$ and $m$ were tallied for each individual, $d$ was tallied for each relationship and $v$ was tallied for each combination between relationship and day.

| Communication System | Variable | Size | min | 2.5%ile | 25%ile | 50%ile | 75%ile | 97.5%ile | max |
|---|---|---|---|---|---|---|---|---|---|
| Twitter | $N$ | 2,585 | 7 | 28 | 65 | 94 | 136 | 264 | 736 |
| | $m$ | 2,585 | 1.25 | 1.84 | 2.79 | 3.55 | 4.66 | 8.61 | 25.23 |
| | $d$ | 278,475 | 1 | 1 | 1 | 1 | 3 | 20 | 166 |
| | $v$ | 943,719 | 2 | 21 | 54 | 94 | 136 | 383 | 14,120 |
| 755 group chat | $N$ | 17,796 | 1 | 1 | 5 | 9 | 17 | 51 | 187 |
| | $m$ | 17,796 | 1.00 | 1.43 | 2.44 | 3.53 | 5.60 | 18.00 | 112.00 |
| | $d$ | 238,611 | 1 | 1 | 1 | 2 | 4 | 18 | 112 |
| | $v$ | 901,212 | 1 | 1 | 17 | 48 | 143 | 1,072 | 31,990 |
| 755 wall communication | $N$ | 20,000 | 1 | 1 | 6 | 11 | 24 | 159 | 1,372 |
| | $m$ | 20,000 | 1.00 | 1.02 | 1.53 | 2.45 | 4.39 | 15.67 | 103.00 |
| | $d$ | 534,475 | 1 | 1 | 1 | 1 | 2 | 13 | 121 |
| | $v$ | 1,270,546 | 3 | 6 | 17 | 33 | 73 | 452 | 17,565 |
| Ameba Pigg | $N$ | 76,379 | 1 | 1 | 7 | 13 | 26 | 86 | 689 |
| | $m$ | 76,379 | 1.00 | 1.11 | 1.91 | 3.14 | 5.82 | 34.50 | 454.00 |
| | $d$ | 1,610,710 | 1 | 1 | 1 | 1 | 3 | 25 | 457 |
| | $v$ | 6,515,626 | 13 | 146 | 365 | 665 | 1,314 | 4,651 | 87,281 |
| Mobile phone (dormitory) | $N$ | 73 | 2 | 16 | 47 | 94 | 126 | 279 | 688 |
| | $m$ | 73 | 1.81 | 2.06 | 3.34 | 3.95 | 4.75 | 7.45 | 8.07 |
| | $d$ | 7,801 | 1 | 1 | 1 | 1 | 2 | 32 | 207 |
| | $v$ | 32,728 | 0 | 0 | 24 | 60 | 223 | 10,261 | 328,031 |
| SMS (dormitory) | $N$ | 48 | 1 | 1 | 4 | 11 | 19 | 194 | 283 |
| | $m$ | 48 | 1.00 | 1.00 | 1.68 | 2.85 | 4.03 | 11.35 | 30.5 |
| | $d$ | 1,233 | 1 | 1 | 1 | 1 | 2 | 30 | 153 |
| | $v$ | 4,942 | 1 | 1 | 1 | 3 | 7 | 36 | 168 |
| E-mail/Letter (Pachur) | $N$ | 39 | 1 | 1 | 5 | 12 | 20 | 59.25 | 64 |
| | $m$ | 39 | 1 | 1 | 1.4 | 1.79 | 2.24 | 3.20 | 3.29 |
| | $d$ | 602 | 1 | 1 | 1 | 1 | 2 | 6.98 | 18 |
| | $v$ | 1,257 | - | - | - | - | - | - | - |
| Mobile phone (friends & family) | $N$ | 114 | 1 | 1 | 6 | 13.5 | 29.75 | 63.18 | 76 |
| | $m$ | 114 | 1 | 1 | 1.64 | 2.17 | 3 | 5.81 | 7.67 |
| | $d$ | 2,264 | 1 | 1 | 1 | 1 | 2 | 20.43 | 88 |
| | $v$ | 6,821 | 1 | 1 | 1 | 1 | 2 | 6 | 251 |
| SMS (friends & family) | $N$ | 109 | 1 | 1 | 6 | 15 | 33 | 59.3 | 113 |
| | $m$ | 109 | 1 | 1 | 1.57 | 4.14 | 7.8 | 14.82 | 24.69 |
| | $d$ | 2,294 | 1 | 1 | 1 | 2 | 5 | 50 | 189 |
| | $v$ | 16,323 | 1 | 1 | 1 | 2 | 3 | 10 | 71 |
| Face to face (Pachur) | $N$ | 39 | 1 | 1.95 | 41 | 56 | 83 | 125.3 | 131 |
| | $m$ | 39 | 1 | 1 | 5.43 | 6.87 | 8.71 | 12.56 | 13.69 |
| | $d$ | 2,370 | 1 | 1 | 1 | 3 | 40.78 | 100 | |
| | $v$ | 17,622 | - | - | - | - | - | - | - |
| Phone (Pachur) | $N$ | 40 | 1 | 4.90 | 11.75 | 16 | 24 | 40.08 | 43 |
| | $m$ | 40 | 1.37 | 1.59 | 2.42 | 3 | 3.97 | 5.44 | 5.95 |
| | $d$ | 733 | 1 | 1 | 1 | 2 | 4 | 15 | 51 |
| | $v$ | 2,565 | - | - | - | - | - | - | - |
| Baboon group A | $N$ | 35 | 1 | 1 | 3.5 | 7 | 11 | 17.45 | 20 |
| | $m$ | 35 | 1 | 1 | 1.23 | 1.67 | 2.26 | 3.03 | 3.91 |
| | $d$ | 266 | 1 | 1 | 1 | 1 | 2 | 8 | 14 |
| | $v$ | 549 | 1 | 1 | 1 | 1 | 2 | 4 | 8 |
| Baboon group B | $N$ | 24 | 1 | 2.15 | 5.75 | 10 | 13 | 16.43 | 17 |
| | $m$ | 24 | 1 | 1 | 1.49 | 1.97 | 2.85 | 4.56 | 4.80 |
| | $d$ | 225 | 1 | 1 | 1 | 2 | 3 | 9.4 | 18 |
| | $v$ | 579 | 1 | 1 | 1 | 1 | 2 | 4 | 8 |

Table 2: Definitions of communication volume $v$.

| Communication System | Definition of $v$ |
|---|---|
| Twitter | Number of characters |
| 755 group chat | Number of characters |
| 755 wall communication | Number of characters |
| Ameba Pigg | Number of characters |
| Mobile phone (dormitory) | Duration |
| SMS (dormitory) | Frequency of messaging |
| E-mail/Letter (Pachur) | Unavailable |
| Mobile phone (friends & family) | Frequency of calls |
| SMS (friends & family) | Frequency of messaging |
| Face to face (Pachur) | Unavailable |
| Phone (Pachur) | Unavailable |
| Baboon group A | Frequency of fur cleaning |
| Baboon group B | Frequency of fur cleaning |
