# Supplementary material for: Two types of social grooming methods depending on the trade-off between the number and strength of social relationships
Source: R Soc Open Sci. 2018 Aug 1;5(8):180148. doi: 10.1098/rsos.180148 (PMC6124085; doi:10.1098/rsos.180148)
Supplement: ESM Section 2 [file rsos180148supp4.pdf]

2 Development of Eq. 1

I develop Eq. 1. The strength of social relationships between i and j at day t ($d_{ij}(t)$) increases the amount of social grooming $v_{ij}(t)$ (Fig. 4). The gradient of this increase depended on a density of social grooming, not frequency (Fig. 5). Thus, v_{ij} does not depend on t, i.e. $d_{ij}(t)/t = w_{ij}$

m_i is the mean of i's strength of social relationships, and N_i and m_i are at time T. That is,

$$m_i = \frac{1}{N_i}\sum_{j=1}^{N_i} d_{ij}(T) = \frac{T}{N_i}\sum_{j=1}^{N_i} w_{ij}. \tag{1}$$

Therefore, I acquire $\sum_{j=1}^{N_i} w_{ij} = \frac{m_i N_i}{T}$.

Here, I used a linear social grooming amount function $v(w_{ij}) = \alpha w_{ij} + 1$ as the simplest assumption. As a result, the total amount of social grooming per day V_i is as follows.

$$V_i = \sum_{j}^{N_i} v(w_{ij}) = \alpha \sum_{j=1}^{N_i} w_{ij} + N_i \tag{2}$$

$$= \alpha m_i N_i/T + N_i \tag{3}$$

Therefore, I acquire a function of the total amount of social grooming per day $V(a, \alpha; N, m) = \alpha mN/T + N$.

$V(a, \alpha; N, m)$ includes reinforcing existing social relationships G (Eq. 1) and making new social relationships G_0. I separate them. Consider an individual $*$ who makes a new social relationship every day and does not reinforce their social relationships, i.e. $C_* = T = N_*$ and $m_* = 1$. Thus, $*$'s $V(a, \alpha; N_*, m_*)T$ is $V_0 N_*$, where V_0 is an amount of social grooming to make a new social relation. This equals $V(a, \alpha; N_*, m_*)T = (\alpha m_* N_*/T + N_*)T$. Therefore, $V_0 = \alpha + T$. As a result, I acquire Eq. 1 in the following.

$$G(a, \alpha; C, m) = \alpha mN/T + N - V_0 N/T \tag{4}$$

$$= \alpha N(m-1)/T \tag{5}$$

$$= \alpha C(m^{1-a} - m^{-a})/T \tag{6}$$

G_0 is $(\alpha + T)N/T \simeq N$, where I consider sufficiently large T, i.e. $T >> \alpha$.
