# Supplementary material for: Two types of social grooming methods depending on the trade-off between the number and strength of social relationships
Source: R Soc Open Sci. 2018 Aug 1;5(8):180148. doi: 10.1098/rsos.180148 (PMC6124085; doi:10.1098/rsos.180148)
Supplement: ESM Section 3 [file rsos180148supp5.pdf]

3 Source Code of the Individual-based Simulations

The following shows an R source code for individual-based simulations with parameters: “alpha” is α, “maxT” is T, “L” is the number of groomers, “N” is vector of groomers’ N, and “m” is vector of groomers’ m. The dimensions of “N” and “m” are “L”. “simulation” function returns each groomer i’s strength of social relationships with each j at T (d_{ij}).

```
library(dplyr)

simulation <- function(alpha, maxT, L, N, m){
  G <- alpha * N * (m-1)/maxT

  res <- tbl_df(bind_rows(lapply(1:L, function(i){
    # expectation value of creating new relationships
    q <- (N[i]-1)/maxT
    # init social relationships
    net <- c(1)

    for(t in 1:maxT){
      # create new relationships
      net <- c(net, rep(1, rpois(1, q)))

      cost <- 0
      done <- rep(0, length(net))

      tmp <- net
      while(G[i] >= cost & sum(done) < length(net)){
        tmp <- net
        tmp[done != 0] <- 0

        p <- cumsum(tmp)/sum(tmp, na.rm=T)
        dart <- runif(1, 0, 1)
        ind <- min(which(p >= dart))

        dcost <- alpha * net[ind]/t + 1

        done[ind] <- 1
        net[ind] <- net[ind] +
                ifelse(cost + dcost > G[i],
                (G[i] - cost) / dcost, 1)

        cost <- cost + dcost
      }

    }
    data.frame(i=i, w=net)
  })))
  res
}
```
