# Supplementary material for: Two types of social grooming methods depending on the trade-off between the number and strength of social relationships
Source: R Soc Open Sci. 2018 Aug 1;5(8):180148. doi: 10.1098/rsos.180148 (PMC6124085; doi:10.1098/rsos.180148)
Supplement: ESM Table 2 [file rsos180148supp6.pdf]

ESM Table 1: The results of the linear regression models in Fig. 7b. The p-values of coefficients of a (β_1) of $k = 1, 2$ were significant (at the 5% level).

k	Coefficient	Estimate	Standard Error	t-value	p-value
1	β_0	0.595164213	0.39365186	1.51190499	1.59×10^{-1}
	β_1	1.539224754	0.37575224	4.09638209	1.77×10^{-3}
2	β_0	1.258377334	0.07185720	17.51219574	2.21×10^{-9}
	β_1	0.177056616	0.06858980	2.58138393	2.55×10^{-2}
3	β_0	1.197881677	0.06372969	18.79628890	1.04×10^{-9}
	β_1	0.040030254	0.06083186	0.65804753	5.24×10^{-1}
4	β_0	1.351159368	0.13209195	10.22893018	5.89×10^{-7}
	β_1	-0.086472250	0.12608564	-0.68582155	5.07×10^{-1}
5	β_0	1.198929874	0.12830630	9.34427939	1.45×10^{-6}
	β_1	-0.017719916	0.12247212	-0.14468530	8.88×10^{-1}
6	β_0	1.234129338	0.12966017	9.51818376	1.21×10^{-6}
	β_1	-0.067735903	0.12376443	-0.54729700	5.95×10^{-1}
7	β_0	1.075730717	0.08512526	12.63703309	6.82×10^{-8}
	β_1	-0.014718476	0.08125456	-0.18114031	8.60×10^{-1}
8	β_0	1.117177430	0.04104771	27.21656140	1.92×10^{-11}
	β_1	-0.029675044	0.03918124	-0.75737889	4.65×10^{-1}
9	β_0	1.202005048	0.08303338	14.47616634	1.66×10^{-8}
	β_1	-0.102764660	0.07925780	-1.29658739	2.21×10^{-1}
10	β_0	1.023633201	0.07217599	14.18246209	2.05×10^{-8}
	β_1	0.024676436	0.06889410	0.35817925	7.27×10^{-1}
11	β_0	1.161198914	0.05956657	19.49413813	7.03×10^{-10}
	β_1	-0.085909899	0.05685804	-1.51095440	1.59×10^{-1}
12	β_0	1.067818465	0.04248356	25.13486474	4.55×10^{-11}
	β_1	-0.023121962	0.04055180	-0.57018336	5.80×10^{-1}
13	β_0	1.041309232	0.04161128	25.02468446	4.77×10^{-11}
	β_1	-0.006097605	0.03971919	-0.15351786	8.80×10^{-1}
14	β_0	1.064526728	0.05579864	19.07800526	3.40×10^{-9}
	β_1	-0.038095942	0.05288340	-0.72037619	4.88×10^{-1}
15	β_0	1.085903589	0.13964035	7.77643148	1.51×10^{-5}
	β_1	-0.002202642	0.13234474	-0.01664322	9.87×10^{-1}
16	β_0	1.074709023	0.04052250	26.52129092	1.34×10^{-10}
	β_1	-0.034052038	0.03840537	-0.88664774	3.96×10^{-1}
17	β_0	1.075165671	0.03367693	31.92587973	2.14×10^{-11}
	β_1	-0.042227567	0.03191746	-1.32302416	2.15×10^{-1}
18	β_0	1.081725558	0.02953836	36.62104214	3.39×10^{-10}
	β_1	-0.040708399	0.02729520	-1.49141240	1.74×10^{-1}
19	β_0	1.039782682	0.03025203	34.37067981	5.61×10^{-10}
	β_1	-0.012328165	0.02795467	-0.44100559	6.71×10^{-1}
20	β_0	1.105641396	0.02986570	37.02044486	3.11×10^{-10}
	β_1	-0.059727472	0.02759768	-2.16422094	6.24×10^{-2}
21	β_0	1.084229708	0.03954838	27.41527576	3.38×10^{-9}
	β_1	-0.042967187	0.03654505	-1.17573205	2.74×10^{-1}
22	β_0	1.128420934	0.04238678	26.62200087	4.26×10^{-9}
	β_1	-0.068939518	0.03916790	-1.76010234	1.16×10^{-1}
23	β_0	1.065976341	0.02828153	37.69160397	2.69×10^{-10}
	β_1	-0.039763309	0.02613382	-1.52152709	1.67×10^{-1}
24	β_0	1.035901088	0.04138359	25.03168837	6.94×10^{-9}
	β_1	-0.023413328	0.03824089	-0.61225891	5.57×10^{-1}
25	β_0	1.047873914	0.03687880	28.41399175	2.54×10^{-9}
	β_1	-0.010192706	0.03407820	-0.29909752	7.72×10^{-1}
26	β_0	1.065386715	0.02064793	51.59775290	2.21×10^{-11}
	β_1	-0.038357512	0.01907991	-2.01036106	7.92×10^{-2}
27	β_0	1.060365867	0.02075569	51.08795346	2.39×10^{-11}
	β_1	-0.033411650	0.01917949	-1.74205075	1.20×10^{-1}
28	β_0	1.032081155	0.02993428	34.47823450	5.47×10^{-10}
	β_1	-0.010874364	0.02766105	-0.39312906	7.04×10^{-1}
29	β_0	1.062367352	0.02777313	38.25162028	2.40×10^{-10}
	β_1	-0.028112242	0.02566402	-1.09539491	3.05×10^{-1}
30	β_0	1.031548375	0.03588988	28.74204403	2.32×10^{-9}
	β_1	-0.022918098	0.03316438	-0.69104567	5.09×10^{-1}
