# Supplementary material for: Two types of social grooming methods depending on the trade-off between the number and strength of social relationships
Source: R Soc Open Sci. 2018 Aug 1;5(8):180148. doi: 10.1098/rsos.180148 (PMC6124085; doi:10.1098/rsos.180148)
Supplement: ESM Table 2 [file rsos180148supp7.pdf]

ESM Table 2: The results of the linear regression models in Fig. 11b. All p-values of coefficients of $a$ ($\beta_1$) were significant (at the 5% level).

| $k$ | Coefficient | Estimate | Standard Error | t-value | p-value |
|---|---|---|---|---|---|
| 1 | $\beta_0$ | -1.332579161 | 0.302972876 | -4.3983448 | $1.34 \times 10^{-4}$ |
| | $\beta_1$ | 5.663778345 | 0.228212373 | 24.8180161 | $4.40 \times 10^{-21}$ |
| 2 | $\beta_0$ | 1.123129400 | 0.027375617 | 41.0266330 | $3.08 \times 10^{-27}$ |
| | $\beta_1$ | 0.768422529 | 0.020620508 | 37.2649663 | $4.77 \times 10^{-26}$ |
| 3 | $\beta_0$ | 1.131964715 | 0.014387637 | 78.6762077 | $2.31 \times 10^{-35}$ |
| | $\beta_1$ | 0.383455157 | 0.010837395 | 35.3825945 | $2.08 \times 10^{-25}$ |
| 4 | $\beta_0$ | 1.112106250 | 0.008726854 | 127.4349524 | $2.03 \times 10^{-41}$ |
| | $\beta_1$ | 0.246792990 | 0.006573447 | 37.5439240 | $3.85 \times 10^{-26}$ |
| 5 | $\beta_0$ | 1.089469269 | 0.009301730 | 117.1254462 | $2.33 \times 10^{-40}$ |
| | $\beta_1$ | 0.185642277 | 0.007006468 | 26.4958420 | $7.13 \times 10^{-22}$ |
| 6 | $\beta_0$ | 1.075891073 | 0.006986234 | 154.0015747 | $8.43 \times 10^{-44}$ |
| | $\beta_1$ | 0.148463744 | 0.005262336 | 28.2125168 | $1.23 \times 10^{-22}$ |
| 7 | $\beta_0$ | 1.064641701 | 0.006144172 | 173.2766801 | $2.77 \times 10^{-45}$ |
| | $\beta_1$ | 0.124857524 | 0.004628058 | 26.9783837 | $4.31 \times 10^{-22}$ |
| 8 | $\beta_0$ | 1.045448912 | 0.009463678 | 110.4696237 | $1.27 \times 10^{-39}$ |
| | $\beta_1$ | 0.116642346 | 0.007128454 | 16.3629223 | $3.49 \times 10^{-16}$ |
| 9 | $\beta_0$ | 1.029676943 | 0.009343060 | 110.2076766 | $1.36 \times 10^{-39}$ |
| | $\beta_1$ | 0.109472850 | 0.007037600 | 15.5554237 | $1.31 \times 10^{-15}$ |
| 10 | $\beta_0$ | 1.023849421 | 0.008476656 | 120.7845905 | $9.58 \times 10^{-41}$ |
| | $\beta_1$ | 0.104584496 | 0.006384987 | 16.3797516 | $3.40 \times 10^{-16}$ |
| 11 | $\beta_0$ | 1.022318131 | 0.011046100 | 92.5501422 | $2.12 \times 10^{-37}$ |
| | $\beta_1$ | 0.096725498 | 0.008320404 | 11.6250965 | $1.95 \times 10^{-12}$ |
| 12 | $\beta_0$ | 1.008509920 | 0.009643737 | 104.5766708 | $6.19 \times 10^{-39}$ |
| | $\beta_1$ | 0.097534645 | 0.007264083 | 13.4269727 | $5.64 \times 10^{-14}$ |
| 13 | $\beta_0$ | 1.007275244 | 0.010372113 | 97.1137930 | $5.27 \times 10^{-38}$ |
| | $\beta_1$ | 0.089559541 | 0.007812728 | 11.4632873 | $2.73 \times 10^{-12}$ |
| 14 | $\beta_0$ | 1.003703991 | 0.011217760 | 89.4745493 | $5.63 \times 10^{-37}$ |
| | $\beta_1$ | 0.085683943 | 0.008449705 | 10.1404651 | $4.80 \times 10^{-11}$ |
| 15 | $\beta_0$ | 0.980619416 | 0.012907327 | 75.9738597 | $6.34 \times 10^{-35}$ |
| | $\beta_1$ | 0.112284086 | 0.009722361 | 11.5490556 | $2.28 \times 10^{-12}$ |
| 16 | $\beta_0$ | 1.005684000 | 0.012076478 | 83.2762647 | $4.48 \times 10^{-36}$ |
| | $\beta_1$ | 0.073467369 | 0.009096530 | 8.0764172 | $6.61 \times 10^{-9}$ |
| 17 | $\beta_0$ | 0.996238636 | 0.013718189 | 72.6217324 | $2.33 \times 10^{-34}$ |
| | $\beta_1$ | 0.076710519 | 0.010333138 | 7.4237393 | $3.52 \times 10^{-8}$ |
| 18 | $\beta_0$ | 0.986484198 | 0.012356490 | 79.8353121 | $1.52 \times 10^{-35}$ |
| | $\beta_1$ | 0.089323920 | 0.009307446 | 9.5970384 | $1.66 \times 10^{-10}$ |
| 19 | $\beta_0$ | 0.998782369 | 0.012540346 | 79.6455162 | $1.62 \times 10^{-35}$ |
| | $\beta_1$ | 0.073269916 | 0.009445935 | 7.7567665 | $1.49 \times 10^{-8}$ |
| 20 | $\beta_0$ | 1.003596599 | 0.014772296 | 67.9377532 | $1.59 \times 10^{-33}$ |
| | $\beta_1$ | 0.066790345 | 0.011127137 | 6.0024735 | $1.58 \times 10^{-6}$ |
| 21 | $\beta_0$ | 0.996122504 | 0.013179879 | 75.5790321 | $7.37 \times 10^{-35}$ |
| | $\beta_1$ | 0.064556907 | 0.009927659 | 6.5027319 | $4.05 \times 10^{-7}$ |
| 22 | $\beta_0$ | 0.963723617 | 0.014097312 | 68.3622227 | $1.33 \times 10^{-33}$ |
| | $\beta_1$ | 0.098058997 | 0.010618710 | 9.2345488 | $3.89 \times 10^{-10}$ |
| 23 | $\beta_0$ | 0.956892921 | 0.017879265 | 53.5196999 | $1.53 \times 10^{-30}$ |
| | $\beta_1$ | 0.103473192 | 0.013467442 | 7.6832106 | $1.80 \times 10^{-8}$ |
| 24 | $\beta_0$ | 0.972817371 | 0.022854669 | 42.5653662 | $1.08 \times 10^{-27}$ |
| | $\beta_1$ | 0.085786524 | 0.017215133 | 4.9832043 | $2.66 \times 10^{-5}$ |
| 25 | $\beta_0$ | 0.987094120 | 0.016874674 | 58.4955974 | $1.19 \times 10^{-31}$ |
| | $\beta_1$ | 0.071439056 | 0.012710740 | 5.6203697 | $4.53 \times 10^{-6}$ |
| 26 | $\beta_0$ | 0.955772717 | 0.015323934 | 62.3712350 | $1.87 \times 10^{-32}$ |
| | $\beta_1$ | 0.103630035 | 0.011542655 | 8.9780066 | $7.18 \times 10^{-10}$ |
| 27 | $\beta_0$ | 0.983677698 | 0.021040709 | 46.7511665 | $7.37 \times 10^{-29}$ |
| | $\beta_1$ | 0.068942098 | 0.015848779 | 4.3499943 | $1.54 \times 10^{-4}$ |
| 28 | $\beta_0$ | 0.971718292 | 0.036132629 | 26.8930968 | $4.71 \times 10^{-22}$ |
| | $\beta_1$ | 0.082356266 | 0.027216671 | 3.0259493 | $5.15 \times 10^{-3}$ |
| 29 | $\beta_0$ | 0.964772033 | 0.025017288 | 38.5642131 | $1.80 \times 10^{-26}$ |
| | $\beta_1$ | 0.085578111 | 0.018844112 | 4.5413714 | $9.06 \times 10^{-5}$ |
| 30 | $\beta_0$ | 0.951722592 | 0.022439565 | 42.4127021 | $1.19 \times 10^{-27}$ |
| | $\beta_1$ | 0.106732294 | 0.016902458 | 6.3146018 | $6.74 \times 10^{-7}$ |
